# Non-thrombotic manifestations in pediatric patients with persistent antiphospholipid antibodies. A Mexican tertiary center case series

**DOI:** 10.3389/fped.2026.1882869

**Published:** 2026-09-23

**Authors:** Meza Luna Ariadna Marisol, García Herrera Iris Paola, Rebolledo Zamora Alejandra, Aparicio Vera Luis Alberto

**Affiliations:** 1 Pediatric Department, Hospital de la Niñez Poblana, Puebla, Puebla; 2 Pediatric Rheumatology Department, Hospital de la Niñez Poblana, Puebla, Puebla; 3 Pediatric Nephrology Department, Hospital de la Niñez Poblana, Puebla, Puebla

**Keywords:** antiphospholipid antibodies, antiphospholipid syndrome—immunology, antiphospholipid antibody syndrome (APS), non-thrombotic antiphospholipid syndrome, pediatric antiphospholipid syndrome

## Abstract

**Introduction:**

Antiphospholipid antibodies (aPL) are a group of autoantibodies classically associated with antiphospholipid syndrome (APS), a disease strongly linked to thrombosis and pregnancy loss. However, non-thrombotic manifestations are increasingly reported, including neurological, hematological, renal, and cardiac involvement. Information is limited at this level. Understanding the role of these antibodies is fundamental to rheumatological practice, especially in the pediatric population where information on this topic is limited.

**Objective:**

To describe non-thrombotic characteristics in pediatric patients with positive antiphospholipid antibodies at a tertiary care hospital in Mexico.

**Material and methods:**

A retrospective, observational, descriptive study was conducted. This study documented non-thrombotic clinical manifestations in pediatric patients with persistently positive antiphospholipid antibodies. Our patients did not meet the diagnostic criteria for antiphospholipid syndrome. Patients with thrombosis were not included in this study. All patients were evaluated by the pediatric rheumatology team.

**Results:**

Eighteen patients were included. Hematological alterations were the most prevalent (72% of patients presented with characteristics, including thrombocytopenia, hemolytic anemia, or leukopenia), followed by neurological manifestations. The most frequent antibody was anti-beta-2-glycoprotein-1, and 22% of patients had a triple positive profile.

**Conclusion:**

The most frequent manifestation reported in our group of patients with positive antiphospholipid antibodies was thrombocytopenia and hematological manifestations in general. The principal antibody reported was the anti-beta-2-glycoprotein-1. We need more studies to get a better understanding about the relation between positive antiphospholipid antibodies and non-thrombotic features.

## Introduction

Antiphospholipid antibodies (aPL), including anticardiolipin antibodies (aCL), anti-beta-2-glycoprotein I antibodies (a*β*2GPI), and lupus anticoagulant (LA), are pathogenic autoantibodies that play a central role in the pathophysiology of antiphospholipid syndrome (APS) and contribute to immune dysregulation in systemic lupus erythematosus (SLE) (1). The presence of these antibodies induces a hypercoagulable state that increases the risk of arterial and venous thrombosis, and obstetric complications such as recurrent pregnancy loss, preeclampsia, and intrauterine growth restriction (2, 3).

Even in patients who do not meet the full APS classification criteria, aPL represent a risk factor for the development of thrombosis or subsequent progression to this syndrome (4). Each antiphospholipid antibody has been described with varying risks for thrombosis and distinct clinical associations. For example, LA is considered the most relevant antibody for thrombosis, followed by a*β*2GPI, whose prothrombotic risk is generally higher in its IgG isotype expression (5).

Transient antiphospholipid antibody positivity has been reported in up to 10%–25% of healthy children, mainly following infections or vaccinations, while persistent positivity occurs in less than 1% of the pediatric population (6).

In a pediatric population thrombosis occurs less frequently, and aPL in children has been associated with various clinical manifestations, including hematologic, neurological, and dermatologic manifestations, even in the absence of thrombosis (7). Understanding the clinical relevance and associations of antiphospholipid antibodies in this age group is crucial to improving early diagnosis, guiding treatment, and preventing complications (8). Despite increasing recognition of non-thrombotic manifestations, their clinical significance and management in pediatric patients remain poorly defined, particularly in Latin American populations.

## Objective

To describe the non-thrombotic characteristics in pediatric patients with positive antiphospholipid antibodies at a Mexican tertiary care hospital.

## Material and methods

A descriptive, observational, retrospective study was conducted on pediatric patients at Hospital para la niñez poblana, a tertiary-care hospital specialized in children in Puebla, Mexico. A database was created with information on all patients with positive antiphospholipid antibodies and non-thrombotic manifestations from 2018 to 2023. The database reported demographic variables such as age and gender. The study population included patients under 18 with non-thrombotic manifestations and positive antiphospholipid antibodies, provided that a manifestation could not be explained by another cause, including infectious, neoplastic, genetic, or inborn errors of immunity. Only those manifestations for which there were previous reports in association with antiphospholipid antibodies were considered. A formal sample size calculation was not performed for this was a retrospective descriptive study. All pediatric patients who met the inclusion criteria and were evaluated at Rheumatology Service during the established time were included. This approach represents a census of all eligible cases identified during the study period. It excluded patients over 18 (Our hospital is a pediatric center; it does not admit adult patients), and those with a prior diagnosis of autoimmune disease (specially systemic lupus erythematosus), insufficient information, or chronic diseases that could explain the symptoms. 3 patients initially presented with hemolytic anemia and thrombocytopenia; however, they tested negative for antiphospholipid antibodies at three months and were therefore excluded. The manifestations under consideration were studied in conjunction with a specialty associated with a predominant organ, such as nephrology, hematology, neurology, or dermatology, so that an approach was comprehensive when evaluating differential diagnoses. The mean follow-up time for the patients was 41.2 months (range: 27–82 months) from the onset of the manifestations to the presentation of this study. The detection of antiphospholipid antibodies included lupus anticoagulant, anticardiolipin (IgG and IgM), and anti-beta-2-glycoprotein (IgG, IgM, and IgA). Positive titers were defined as those >40 U/mL or above the 99th percentile, and positivity was considered persistent if the patient had more than one positive determination, with a minimum interval of 12 weeks between each determination. Anticardiolipin and anti-beta-2-glycoprotein antibodies were measured using ELISA, and lupus anticoagulant was determined according to the recommendations of the International Society on Thrombosis and Haemostasis (Three steps procedure: screening, mixing and confirmation. Using Russell's viper venom and activated partial thromboplastin time) (9). Epidemiological variables and clinical characteristics were described using descriptive statistics and frequencies with a 95% confidence interval (calculated using binomial exact methods). All patients were evaluated for antinuclear antibodies (ANA), complement C3, complement C4, anti-dsDNA, and anti-Sm, anti-Ro, anti-La, and anti-RNP. Infectious serologies or bone marrow aspirations were also performed if a clinical presentation required the intentional exclusion of infectious or oncological etiologies. Our patients did not meet the diagnostic criteria for antiphospholipid syndrome. Patients with thrombosis were obviously not included. All patients were evaluated by a pediatric rheumatology clinic.

## Results

Eighteen patients were included from 2019 to 2023. Themean age at diagnosis was 14.4 years old. Fourteen (77.7%) were female, and all patients were Mexican. The non-thrombotic manifestations found were thrombocytopenia, hemolytic anemia, leukopenia and lymphopenia, hallucinations, hearing loss, seizures, Guillain-Barré syndrome, episcleritis, nephrotic syndrome, and Raynaud's phenomenon.

Within our population, hematological manifestations were the most frequent. They were observed in 13 patients (72.2%; 95% CI: 46.5–90.3), followed by neurological involvement in 3 patients (16.6%; 95% CI: 3.6–41.4) (Table 1, Figure 1). Raynaud's phenomenon, hearing loss, nephrotic syndrome, and episcleritis were each reported in only one patient. The patient with nephrotic syndrome could not undergo a kidney biopsy.

**Table 1:** Clinical and serological features in patients with positive antiphospholipid antibodies and non-thrombotic disease.

Gender	Age (Years)	Non-trhombotic manifestations	Associated positive antibodies	Positive antibodies after 12 weks
Male	6	Thrombocytopenia	IgA Anti-beta-2-Glycoprotein-1	IgA Anti-beta-2-Glycoprotein-1
Female	16	Thrombocytopenia and metrorrhagia	Lupus anticoagulant. IgG Anticardiolipin, IgA and IgG Anti-beta-2-Glycoprotein-1	Lupus anticoagulant. IgA and IgG Anti-beta-2-Glycoprotein-1
Female	6	Thrombocytopenia	IgM Anticardiolipin	IgM Anticardiolipin
Female	15	Thrombocytopenia	IgG, IgM and IgA Anti-beta-2-Glycoprotein-1 IgG and IgM Anticardiolipin, Lupus anticoagulant.	IgG, IgM and IgA Anti-beta-2-Glycoprotein-1 Lupus anticoagulant.
Female	15	Auditory hallucinations.	Anti-beta-2-Glycoprotein-1	Anti-beta-2-Glycoprotein-1
Female	12	Bilateral sensorineural hearing loss.	Lupus anticoagulant.	Lupus anticoagulant.
Male	14	Autoimmune hemolytic anemia and thrombocytopenia.	IgM Anti-beta-2-Glycoprotein-1 IgM Anticardiolipin	IgM Anti-beta-2-Glycoprotein-1 IgM Anticardiolipin
Female	13	Leukolymphopenia Thrombocytopenia Visual hallucinations, seizures.	Anti-beta-2-Glycoprotein-1	Anti-beta-2-Glycoprotein-1
Female	15	Thrombocytopenia.	IgA Anti-beta-2-Glycoprotein-1, IgM Anticardiolipin	IgA Anti-beta-2-Glycoprotein-1
Female	7	Autoimmune hemolytic anemia.	IgA Anti-beta-2-Glycoprotein-1, IgM Anticardiolipin, Lupus anticoagulant	IgA Anti-beta-2-Glycoprotein-1, Lupus anticoagulant
Male	6	Guillain-Barre syndrome.	IgM Anti-beta-2-Glycoprotein-1, IgG and IgM Anticardiolipin	IgM Anti-beta-2-Glycoprotein-1,
Female	13	Leukopenia. Raynaud's phenomenon	IgM Anti-beta-2-Glycoprotein-1, Lupus anticoagulant.	IgM Anti-beta-2-Glycoprotein-1.
Male	15	Leukopenia and thrombocytopenia.	IgA Anti-beta-2-Glycoprotein-1. IgG Anticardiolipin	IgA Anti-beta-2-Glycoprotein-1. IgG Anticardiolipin
Female	5	Nephrotic syndrome.	IgG e IgM Anti-beta-2-Glycoprotein-1, IgM Anticardiolipin Lupus anticoagulant	IgG e IgM Anti-beta-2-Glycoprotein-1, IgM Anticardiolipin
Female	11	Leukopenia Thrombocytopenia	IgG and IgM Anticardiolipin Lupus anticoagulant	Lupus anticoagulant
Female	9	Nodular episcleritis	IgG and IgA Anti-beta-2-Glycoprotein-1 IgM Anticardiolipin	IgG and IgA Anti-beta-2-Glycoprotein-1 IgM Anticardiolipin
Female	15	Thrombocytopenia	IgM and IgA Anti-beta-2-Glycoprotein-1 IgM Anticardiolipin	IgM and IgA Anti-beta-2-Glycoprotein-1 IgM Anticardiolipin
Female	13	Thrombocytopenia	IgG, IgA and IgM Anti-beta-2-Glycoprotein-1 IgG Anticardiolipin	IgG, IgA and IgM Anti-beta-2-Glycoprotein-1 IgG Anticardiolipin

Anticardiolipin antibodies and anti-beta-2-Glycoprotein were determinated by Elisa. Lupus anticoagulant was determinated by according to the recommendations of the International Society on Thrombosis and Haemostasis (12). Positive for Anticardiolipin and Anti-beta-2-Glycoprotein were considered with >40 U/mL or above the 99th percentile.

**Figure 1:**
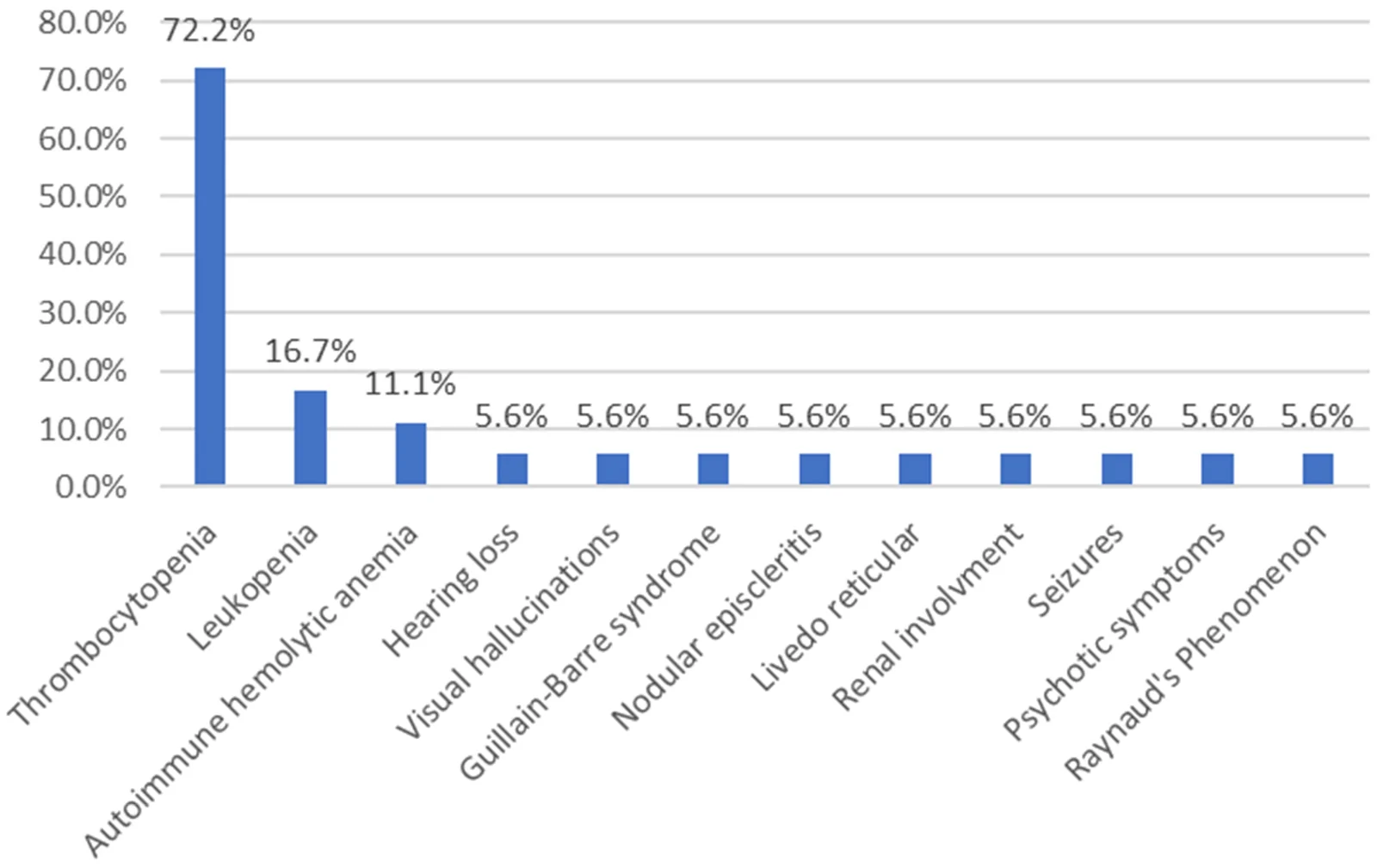
Non-thrombotic manifestations in patients with antiphospholipid antibodies. Type of manifestation and frequency of presentation. The graph represents the total population; however, the sum does not equal 100% because some patients presented with more than one manifestation. Leukopenia was defined as a white blood cell count <5.5 × 10³/µL, anemia as hemoglobin <11.0 g/dL, and thrombocytopenia as a platelet count <150 × 10³/µL, according to the institutional laboratory reference ranges used for the study cohort.

Regarding treatment, azathioprine, hydroxychloroquine, and systemic corticosteroids (prednisone) were used as first-line therapy for both hematological and neurological manifestations. A patient with Guillain-Barré syndrome received standard treatment with intravenous immunoglobulin (2 g/kg). All patients received antiplatelet therapy with aspirin; however, anticoagulant therapy (warfarin or acenocoumarol) was reserved for selected cases, including those with mild to moderate thrombocytopenia, episcleritis, hearing loss, and nephrotic syndrome. Associated manifestations (such as a need for hearing aids, blood pressure control, neurological rehabilitation, anticonvulsant treatment, as well as hematological, renal, and ophthalmological management) were closely monitored and treated using a multidisciplinary approach, involving hematology, nephrology, neurology, and audiology. The patient with hearing loss ultimately required cochlear implantation.

Immunological profiles showed positivity for anti-*β*2-glycoprotein I antibodies in 15 patients (83.3%; 95% CI: 58.6%–94.6%), anticardiolipin antibodies in 12 patients (66%), and lupus anticoagulant in 7 patients (38.8%). Notably, five patients (27.7%; 95% CI: 9.7%–53.5%) had triple-positive serology for antiphospholipid antibodies (anticardiolipin, anti-*β*2-glycoprotein, and lupus anticoagulant); interestingly, the same number of patients (*n* = 5; 27.7%) had only one positive antibody (Figures 2, 3). With the GBS case the diagnostic approach considered other conditions, such as disorder within the spectrum of autoimmune demyelinating diseases; MRI findings were unremarkable, and cerebrospinal fluid tested negative for oligloclonal bands and aquaporin-4.

**Figure 2:**
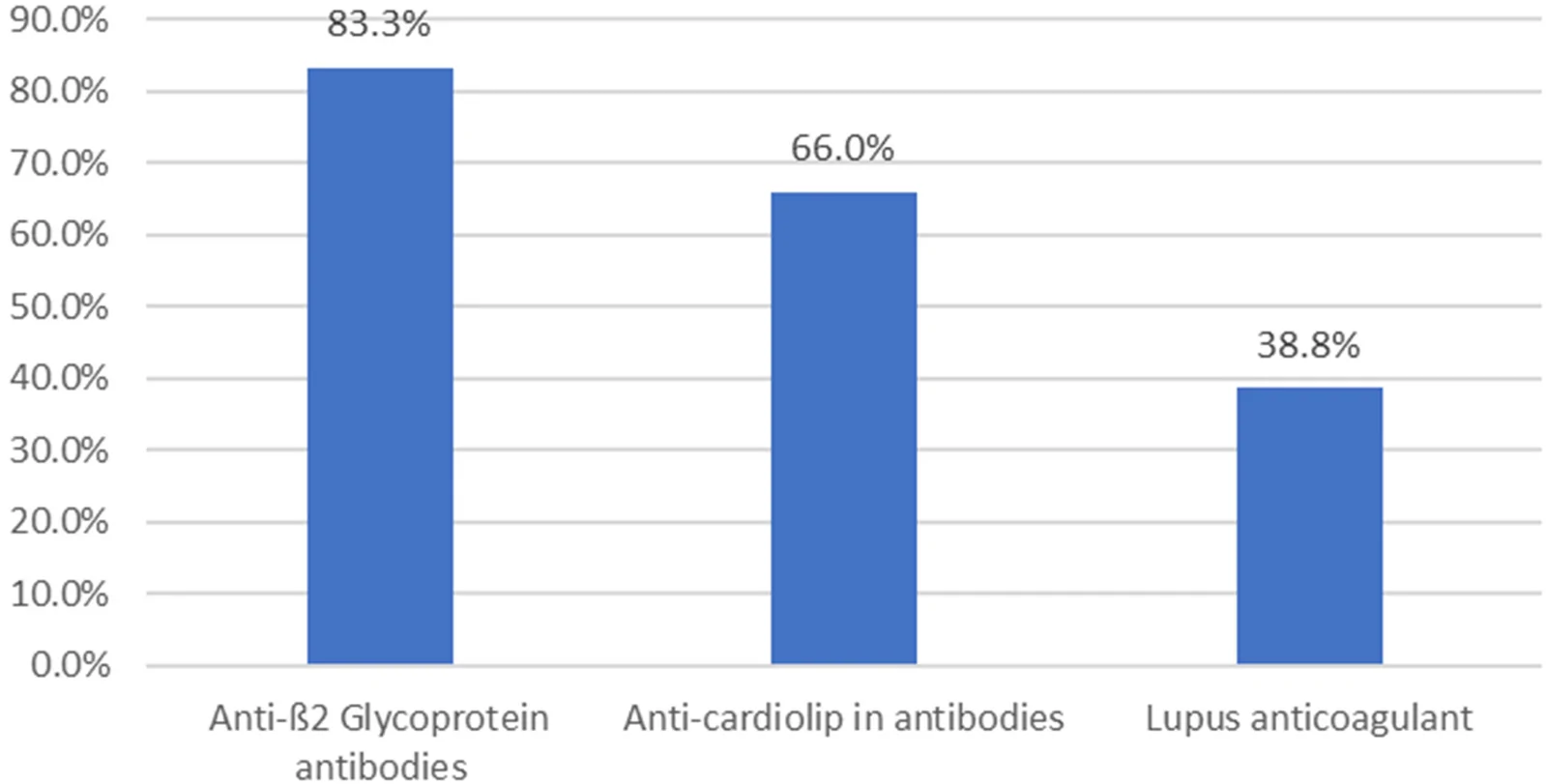
Antibodies distribution between all the pediatric population. *The graph represents the total population, however, it does not equal 100% in the sum, because some patients presented with more than one positive antibodie.

**Figure 3:**
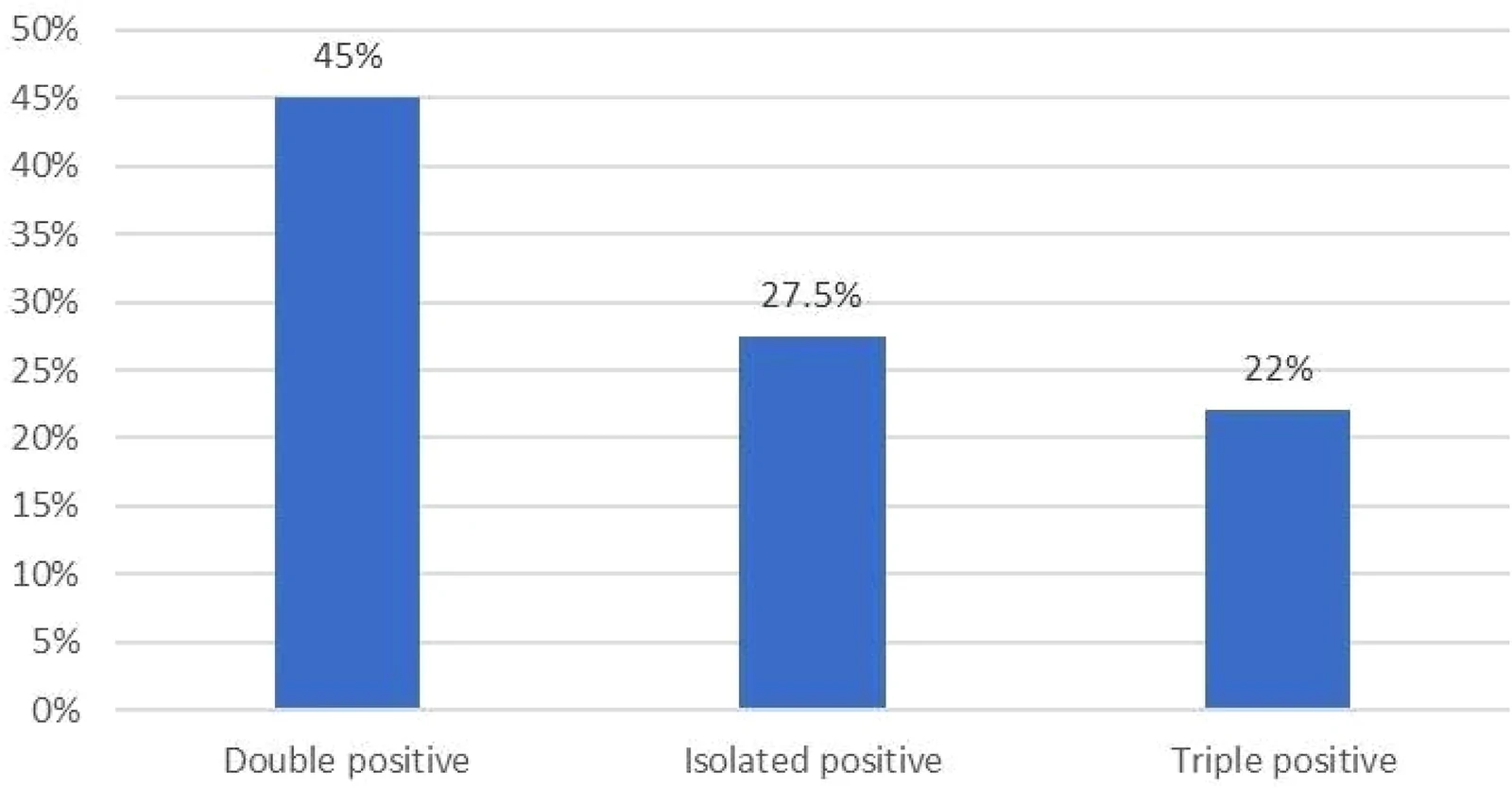
Serological distribution considering the positive amount of antibodies at every patient.

## Discussion

Antiphospholipid syndrome (APS) is a rare autoimmune disorder in children, with an estimated incidence of 0.3–0.7 per 100,000 children per year and a prevalence of 1–5 per 10,000 pediatric patients (10). According to current APS classification systems, including the updated 2023 ACR/EULAR criteria, classification requires clinical and laboratory evidence, with positive antiphospholipid antibodies (4). However, applying these criteria in children is often complex due to a lack of obstetric features and a lower thrombosis incidence of thrombosis in pediatric populations (5), which can delay recognition of the disease. The ACR/EULAR (American College of Rheumatology/European Alliance of Associations for Rheumatology) classification criteria for antiphospholipid syndrome include non-thrombotic clinical domains, such as hematologic, cardiac, and microvascular manifestations, so pediatric patients with non-thrombotic or obstetric morbidity could be classified (4).

Recent studies have shown a high prevalence of non-thrombotic manifestations in children with aPL. According to the International Pediatric APS Registry, non-thrombotic clinical manifestations associated with primary APS included hematologic manifestations (38%), cutaneous disorders (18%), and neurologic manifestations (16%) (6). Positivity for aCL, a*β*2GPI, and AL was found in 81%, 67%, and 72% of patients, respectively. In a previous Mexican report (2012), nonthrombotic hematological and neurological disorders were observed in 53% and 22% of patients with primary APS (7). Ten years later, non-thrombotic features in Mexican children included thrombocytopenia in 69%, autoimmune hemolytic anemia in 40%, Raynaud's phenomenon in 25%, and renal involvement in 13% (8). According to our results, hematological manifestations were also prominent. Although there was a difference in the percentage of other manifestations compared to those reported in the International Pediatric APS Registry and in the Mexican study mentioned above, the prevalence of thrombocytopenia remained higher than other manifestations.

“Extra-criteria” manifestations encompass additional manifestations that can affect multiple organs and cannot be explained solely by a thrombotic mechanism (9). However, there is insufficient literature on “extra-criteria” manifestations in this population. In our cohort, hematological abnormalities were the most frequent manifestations, particularly thrombocytopenia (72.2%). This pattern is consistent with previous pediatric series of antiphospholipid syndrome (APS), which consistently highlight blood cell abnormalities as the predominant presentation in children with persistently positive antiphospholipid antibodies. aPL testing was not performed in cases of acute immune thrombocytopenia (ITP), and testing was only conducted in children with chronic thrombocytopenia. Previous studies have shown that a substantial proportion of pediatric ITP cases exhibit transient aPL positivity (25%–77%), often related to post-infectious immune activation (11, 12). In contrast, our patients presented with persistent antibody positivity and negative infectious serology, supporting a pathophysiological association with aPL rather than transient post-infectious changes. Thrombocytopenia is a significant finding in antiphospholipid syndrome and is one of the most frequently reported non-thrombotic manifestations in patients with positive antiphospholipid antibodies (15). Thrombocytopenia is even more common in the pediatric population than in adults. Erton et al, report data from APS ACTION (AntiPhospholipid Syndrome Alliance for Clinical Trials and InternatiOnal Networking): Of 1,039 persistently aPL-positive patients included in the registry, 228 (22%) were reported to have TP and/or AIHA ever (TP only: 176 (17%). Of 1,039 patients, 59% had primary aPL with or without APS classification (16).

Platelets are increasingly recognized for their functions beyond hemostasis and thrombosis. The presence of cell aggregates formed by the binding of platelets to leukocytes has been reported in patients with antiphospholipid syndrome, which may reflect persistent platelet activation (13, 14). This state of platelet activation can increase platelet binding to antiphospholipid antibodies, impair platelet clearance, or promote the formation of immune complexes. This results in an association between platelet consumption and antiphospholipid antibody activity. Thrombocytopenia in APS is usually mild, and platelet counts are generally maintained above 40 × 10⁹/L. Severe thrombocytopenia associated with APS is managed similarly to primary ITP, using glucocorticoids and intravenous immunoglobulin (16). Second-line treatments include immunosuppressive agents such as azathioprine, cyclophosphamide and rituximab (22). Patients with APS and thrombocytopenia may receive antiplatelet agents and/or anticoagulant therapy. However, anticoagulant therapy remains controversial, despite the known increased thrombotic risk associated with thrombocytopenia in APS (17, 18). The term “aPL-associated thrombocytopenia” is appropriate for patients with thrombocytopenia who do not meet the defining criteria for APS (8).

Among our patients, neurological manifestations were the second most frequent, including various manifestations such as hallucinations, seizures, hearing loss, and Guillain-Barré syndrome. Previous studies on neurological involvement in APS describe thrombotic and non-thrombotic manifestations, such as epilepsy, dementia, chorea, multiple sclerosis-like syndromes, Guillain-Barré syndrome, and bilateral sensorineural hearing loss (19). Psychiatric disorders, including psychosis and schizophrenia-like symptoms, have also been reported in patients with antiphospholipid antibodies (20–23). In APS-associated psychosis, delusions and hallucinations are common, and reported outcomes are generally favorable (23). Some neurological symptoms cannot be explained solely by thrombosis or a hypercoagulable state, suggesting direct effects of antiphospholipid antibodies on neurons or glial cells. Although the vulnerability of the central nervous system in patients with APS is not fully understood, animal models demonstrate that antiphospholipid antibodies (aPL) can bind to neurons, glial cells, and myelin (24, 36). Other manifestations, such as hearing loss or Guillain-Barré syndrome, have been described mainly in case reports, which limits understanding of their pathogenic mechanisms (25). There is a lack of formal recommendations for the management of these patients, and current practice is largely based on expert consensus.

Renal involvement is a well-recognized complication of antiphospholipid syndrome, whether primary or associated with other rheumatic diseases. Renal injury in APS results from thrombosis of the renal artery or vein and microvascular damage known as APS-associated nephropathy (26, 27). Clinical manifestations include varying degrees of proteinuria, hypertension, hematuria, acute kidney injury, and chronic kidney disease. Characteristic renal biopsy findings may involve vessels, veins, and arteries, from the renal arteries to the glomerular capillaries, and classically include arteriosclerosis, fibrous intimal hyperplasia, fibrocellular or arteriolar occlusion, and microangiopathy. In adults, the association between APS and other glomerular lesions is increasingly recognized (28–31). Fakhuri et al. reported membranous nephropathy, minimal change disease, focal segmental glomerulosclerosis, C3 mesangial nephropathy, and pauci-immune glomerulonephritis (28); Immunofluorescence was negative in 66% of cases and positive for C3 and IgG (subepithelial) in the remaining patients. Similarly, Sinico et al. also found predominantly membranous nephropathy, although proliferative glomerulonephritis was also described (32). However, current evidence is insufficient to attribute these findings to a non-thrombotic mechanism of APS. Our patient presented with steroid-dependent nephrotic syndrome with focal segmental glomerulosclerosis, without vascular involvement, microangiopathy, or positive immunofluorescence. After two years of follow-up, no additional immunological disease has been identified. As mentioned previously, these findings may be explained by a primary glomerulopathy, although a renal manifestation associated with aPL cannot be excluded. Pediatric literature remains sparse, with only a few cases reported, including cases of focal proliferative glomerulonephritis and mesangial proliferative glomerulonephritis reported by Minisola et al. and Ohtomo et al., respectively (33, 34). There are no specific therapeutic recommendations for pediatric APS patients without thrombosis; immunosuppressants and anticoagulation have been used with variable results. Our patient achieved partial remission with mycophenolate mofetil and low-dose steroids. It is recommended that all patients with APS-associated nephropathy be evaluated for secondary causes, such as systemic lupus erythematosus (SLE), and that a renal biopsy be performed to confirm the diagnosis (26). However, in patients with aPL without full APS criteria and with renal involvement, the diagnosis can be challenging, and treatment should be individualized.

Current recommendations to treat pediatric antiphospholipid syndrome were published in 2016 by the SHARE initiative (Single Hub and Access point for pediatric Rheumatology in Europe). These guidelines emphasize the importance of individualized management in children with APS or persistent aPL positivity (35). However, there is no consensus on thrombosis prevention or specific therapeutic strategies in patients with persistent aPL positivity without thrombosis. Primary thrombosis prevention should therefore be defined individually in high-risk aPL patients, guided by regular monitoring of antibody titers and strict control of modifiable thrombotic risk factors.

The estimated risk of thrombosis in aPL-positive individuals without prior events or additional risk factors is <1% per year, making the routine use of heparin or aspirin controversial (37, 38). Nevertheless, the need for targeted preventive options arises from the known increased thrombotic risk associated with persistent aPL positivity.

Hydroxychloroquine (HCQ) has demonstrated anti-inflammatory and antithrombotic effects that may help reduce the risk of thrombosis in individuals with antiphospholipid antibodies (39, 40). Although current guidelines recommend its use only in patients with systemic lupus erythematosus (SLE) and aPL positivity, growing evidence supports its potential as an adjunctive therapy in selected pediatric cases with clinical manifestations. Further prospective studies are needed to clarify its prophylactic role in this context.

Few studies have evaluated primary prevention in aPL-positive patients. One study comparing patients with antiphospholipid syndrome (APS) and aPL-positive subjects without thrombosis found that aspirin and/or HCQ use was associated with a lower risk of thrombosis according to logistic regression analysis (39). Despite these findings, current recommendations continue to support HCQ use only in patients with SLE and aPL positivity. We consider HCQ use to be reasonable as adjunctive therapy in aPL-positive patients with clinical symptoms. However, further prospective studies are needed. The role of additional immunomodulatory therapies in APS remains under debate. Agents such as eculizumab and sirolimus are other options for complicated APS.

In children with SLE and aPL positivity, antiplatelet agents may be considered for primary thrombosis prevention in conjunction with HCQ (41, 42). However, long-term aspirin prophylaxis is not recommended in asymptomatic children, and decisions should be individualized based on the aPL profile and the presence of additional acquired or congenital prothrombotic factors.

This study has several limitations that should be acknowledged. First, the small sample size limits the generalizability of the findings and reduces the ability to establish robust associations between antiphospholipid antibodies and specific non-thrombotic manifestations. Second, the retrospective design may introduce information bias, as data collection depended on the accuracy and completeness of medical records. Third, this was a single-center study conducted at a tertiary referral hospital, which may result in selection bias and overrepresentation of more complex or severe cases. Additionally, patients were primarily evaluated in a rheumatology setting, which could further influence the clinical spectrum observed. Although efforts were made to exclude alternative causes of the observed manifestations, residual confounding cannot be completely ruled out, particularly for conditions such as hematological or neurological abnormalities that may have multifactorial etiologies.

Finally, the heterogeneity of clinical manifestations and the lack of standardized definitions for some non-thrombotic features may affect the reproducibility of the findings. Prospective, multicenter studies with larger sample sizes and standardized outcome definitions are needed to better characterize the clinical significance of non-thrombotic manifestations in pediatric patients with antiphospholipid antibodies.

## Conclusion

Non-thrombotic manifestations associated with antiphospholipid antibodies in pediatric patients are diverse, and in many cases, represent the first or only clinical presentation. Hematological and neurological were the most frequently observed in our series. Targeted screening for antiphospholipid antibodies should be performed in children with these manifestations. Further studies are needed to establish a prothrombotic risk in patients with antiphospholipid antibodies who present with non-thrombotic manifestations.

## Data Availability

The original contributions presented in the study are included in the article/Supplementary Material, further inquiries can be directed to the corresponding author.
